# Supplementary material for: Nitrogen-Doped Bismuth Nanosheet as an Efficient Electrocatalyst to CO2 Reduction for Production of Formate
Source: Int J Mol Sci. 2022 Nov 21;23(22):14485. doi: 10.3390/ijms232214485 (PMC9697466; doi:10.3390/ijms232214485)
Supplement: Supplementary file 1 [file ijms-23-14485-s001.zip › ijms-2042392-supplementary.pdf]

Supplementary information

Nitrogen-doped Bismuth Nanosheet as an Efficient Electrocatalyst to CO_2 Reduction for Production of Formate

Sanxiu Li[1,†], Yufei Kang[1,†], Chenyang Mo[1], Yage Peng[1], Haijun Ma[2], Juan Peng[1,*]

[1] State Key Laboratory of High−Efficiency Utilization of Coal and Green Chemical Engineering, College of Chemistry and Chemical Engineering, Ningxia University, Yinchuan 750021, China

[2] Key Laboratory of Ministry of Education for Protection and Utilization of Special Biological Resources in Western China, School of Life Sciences, Ningxia University, Yinchuan 750021, China

∗ Correspondence: pengjuan@nxu.edu.cn

[†]The authors contribute equally to this work.

Chemicals and Reagents

The following chemicals were purchased and used without further purification: Bismuth citrate (purity⩾99.9%) powder was purchased from the MACKLIN reagent network in China. Calcium hydroxide (purity⩾95%, $Ca(OH)_2$) and ammonium chloride (purity⩾99.5%, NH_4Cl) were obtained from Tianjin Shengao Chemical Reagent Co. , Ltd. in China. Nafion solution (5.0 wt%) was obtained from Tianjin Incole Union Technology Co .,Ltd in China. Hydrochloric acid (36.0 – 38.0%, HCl) , potassium bicarbonate (purity ⩾99.5%, $KHCO_3$) was purchased from Sinopharm Chemical Reagent Co., Ltd in China. Self-produced Deionized water. (18.24 MΩ cm^{-1}) was used in the entire experiment.

Characterization and measurement

Powder X-ray diffraction (XRD) measurements were conducted by a Smart Target X-ray diffractometer (Smart Lab, Japan). To observe the surface morphology of each electrocatalyst, we used an F-SEM cold field emission scanning electron microscope (F-SEM, Zeiss Sigma 300, Germany). High-resolution transmission electron microscope (HRTEM) and a high angle annular dark-field scanning transmission electron microscope (HAADFSTEM) was also used to obtain high-resolution images (FEI Talos F200x, America). Elemental content was analyzed by X-ray photoelectron spectroscopy (XPS, Thermo Scientific K-alpha, America), using a monochromatic Al-Kα radiation source (Mono Al-Kα) with 1486.6 eV of energy. Chemical states and surface material composition an X-ray photoelectron spectrometer was used for charge neutralization in the range of 75-150W with a low energy electron gun using a single cathode light source in a super-vacuum state. For the samples before electrocatalytic reaction, we directly characterize the freshly prepared electrodes. For the sample after electrocatalytic reaction, we washed the sample with deionized water, removed the surface electrolyte, and carried out characterization after drying.

Density Functional Theory Simulation

The free energies of CO_2 reduction states were carried out by the Vienna Ab-initio Simulation Package (VASP)[1, 2], taking advantage of the density

functional theory (DFT) with the Projected Augmented Wave[3] (PAW) method. The revised Perdew-Burke-Ernzerhof (RPBE) functional was used to describe the exchange and correlation effects[4-6]. For all the geometry optimizations, the cutoff energy was set to be 500 eV. The Monkhorst-Pack grids[7] were set to be 3×2×1 for performing the surface calculations. The (012) surface of Bi was used to represent the catalytic surface. The slab models were constructed by using four atomic layers. The nitrogen dopant was introduced by substituting surface bismuth atom. During the calculations, the bottom two layers were kept fixed, while the top two layers and the adsorbates were allowed to fully relax. A 15 Å vacuum thickness was added in the z-direction of the simulation box, preventing the interactions between the adjacent slabs.

In aqueous conditions, the reduction of CO_2 to produce HCOOH could occur in the following two elementary steps:

$CO_2 + (H+ + e–) + * \rightarrow *OCHO$

$*OCHO + (H+ + e–) \rightarrow * + HCOOH$

Based on the above mechanism, the free energy of *OCHO intermediate is important to identify a given material's activity in catalysing CO_2 reduction. The computational hydrogen electrode[8] (CHE) model proposed by Norskov et al. was used to calculate the free energies of CO_2 reduction intermediates, based on which the free energy of an adsorbed species is defined as

$$\Delta G_{ads} = \Delta E_{ads} + \Delta E_{ZPE} - T\Delta S_{ads} + \int C_P dT$$

Where ΔE_{ads} is the electronic adsorption energy, ΔE_{ZPE} is the zero point energy difference between adsorbed and gaseous species, $T\Delta S_{ads}$ is the corresponding entropy difference between these two states, and $\int C_P dT$ is the enthalpy correction. The electronic binding energy is referenced as graphene for each C atom, ½ H_2 for each H atom, and (H_2O – H_2) for each O atom, plus the energy of the clean slab.

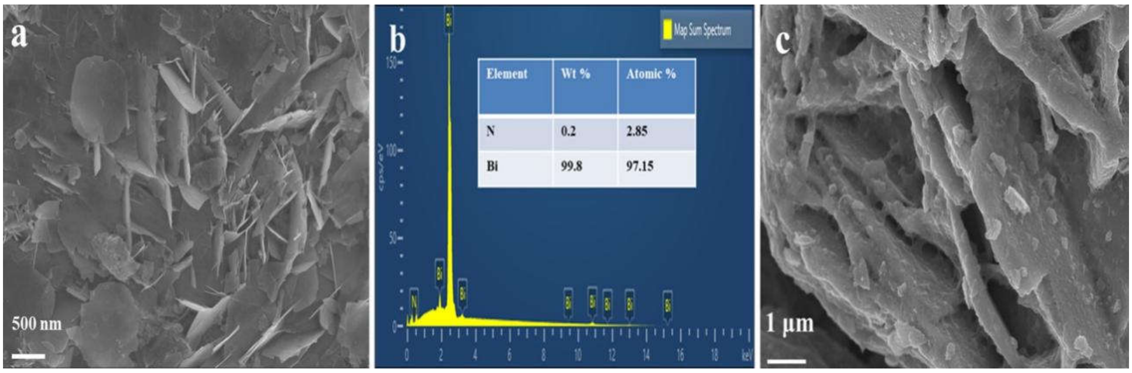

Figure S1. (a) SEM image of N−BiNSs , (b) SEM−EDS of N−BiNSs, (c) SEM image of BiNSs.

Table S1. Performance comparison of N−Bi nanosheets for electrocatalytic CO_2RR towards foamate production with other representative electrocatalysts in the literatures

Electrocatalyst	Electrolyte	Potential	$FE_{formate}$ (%)	Current density	Ref.
N−BiNSs	0.5 M $KHCO_3$	-0.95 V (Vvs. RHE)	94.27	33.63 mA cm^{-2}	This work
BiNSs			81.54	10.29 mA cm^{-2}	
Ultrathin Bi nanosheets	0.1 M $KHCO_3$	-1.10 V (vs. RHE)	90.1	16.5 mA cm^{-2}	[9]
Bi@Sn NPs	0.5 M $KHCO_3$	-1.10 V (vs. RHE)	91	31.0 mA cm^{-2}	[10]
In_2O_3–ZnO NCs	0.5 M $KHCO_3$	-1.20 V (vs. RHE)	.95	22 mA cm-^{2}	[11]
SnO_x/C	0.5 M $KHCO_3$	-0.75V (vs. RHE)	84.2	6.7 mA cm^{-2}	[12]
Bi-Sn	0.1 M $KHCO_3$	-1.0 V (vs. RHE)	93.9	12.4 mA cm^{-2}	[13]
Sb nanosheets	0.5 M $NaHCO_3$	-1.06 V (vs. RHE)	84.0	9.5 mA cm^{-2}	[14]
$Cu_{57}Sn_{43}$	0.05 M $KHCO_3$	-0.92 V (vs. RHE)	57.0	2.5 mA cm^{-2}	[15]
Bi	0.1 M $KHCO_3$	-0.79 V (vs. RHE)	20.0	--	[16]
B doped graphene			66.0	--	
Sn dendrite electrode	0.1 M $KHCO_3$	-1.36 V (vs. RHE)	71.6	17.1 mA cm^{-2}	[17]
Sn/CNT–Agls/CC	0.5 M $KHCO_3$	−0.96 V (vs. RHE)	82.7	26.7 mA cm^{-2}	[18]
P-orbital localized–Bi	0.5 M $KHCO_3$	-1.160 V (vs. RHE)	95	54.1 mA cm^{-2}	[19]
SnO_2 pNWs	0.1 M $NaHCO_3$	-0.8 V (V vs. RHE)	80	4.8 mA cm^{-2}	[20]
Ultrasmall SnO_2 NP	1 M $KHCO_3$	-1.21 V (vs. RHE)	64	92.8 mA cm^{-2}	[21]
BiO_x/C	0.5 M $NaHCO_3$	-1.37 V (vs. Ag/AgCl)	92.1	1.35 mA cm^{-2}	[22]
Sulfide-derived Bi	0.5 M $NaHCO_3$	-0.75 V (vs. RHE)	84	4.2 mA cm^{-2}	[23]
Sn-CF1000	0.1 M $KHCO_3$	-0.8V (vs. RHE)	62	11 mA cm^{-2}	[24]

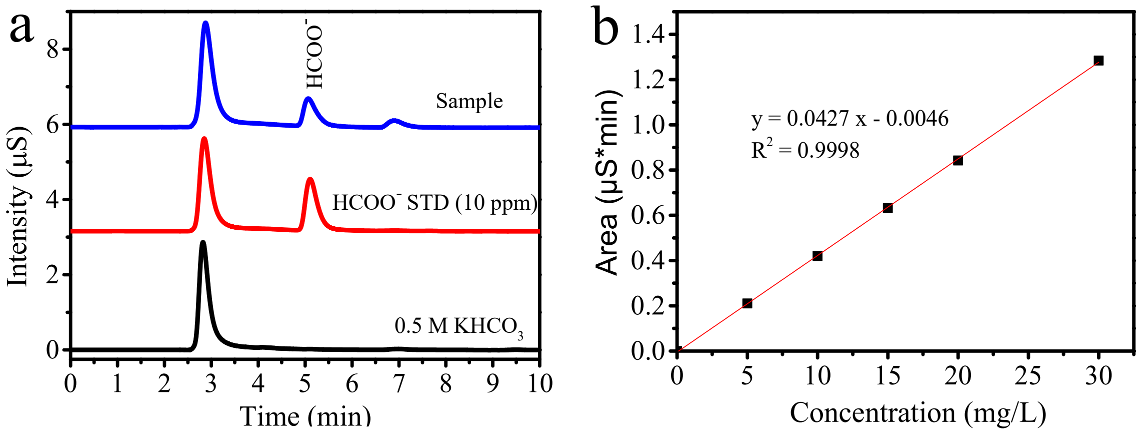

Figure S2. (a) The formate was detected in the reduction product by IC. (b) Linear diagram of peak area and concentration.

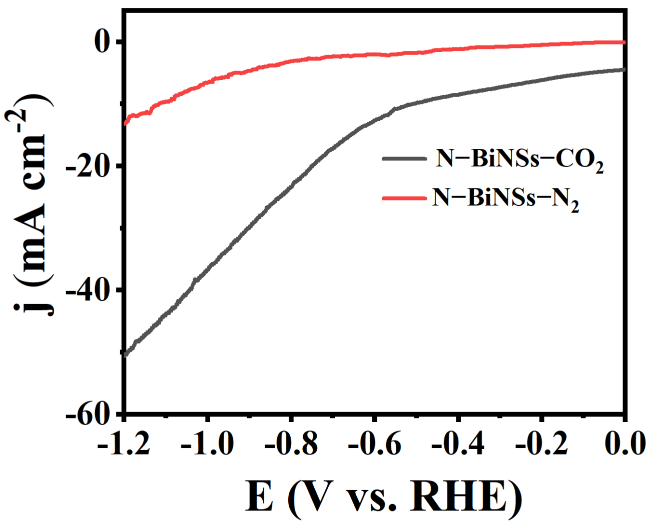

Figure S3. The LSV of N−BiNSs catalyst in 0.5 M $KHCO_3$ aqueous solutions with saturated gases N_2 or CO_2, sweeping speed of 10 mV s^{-1}.

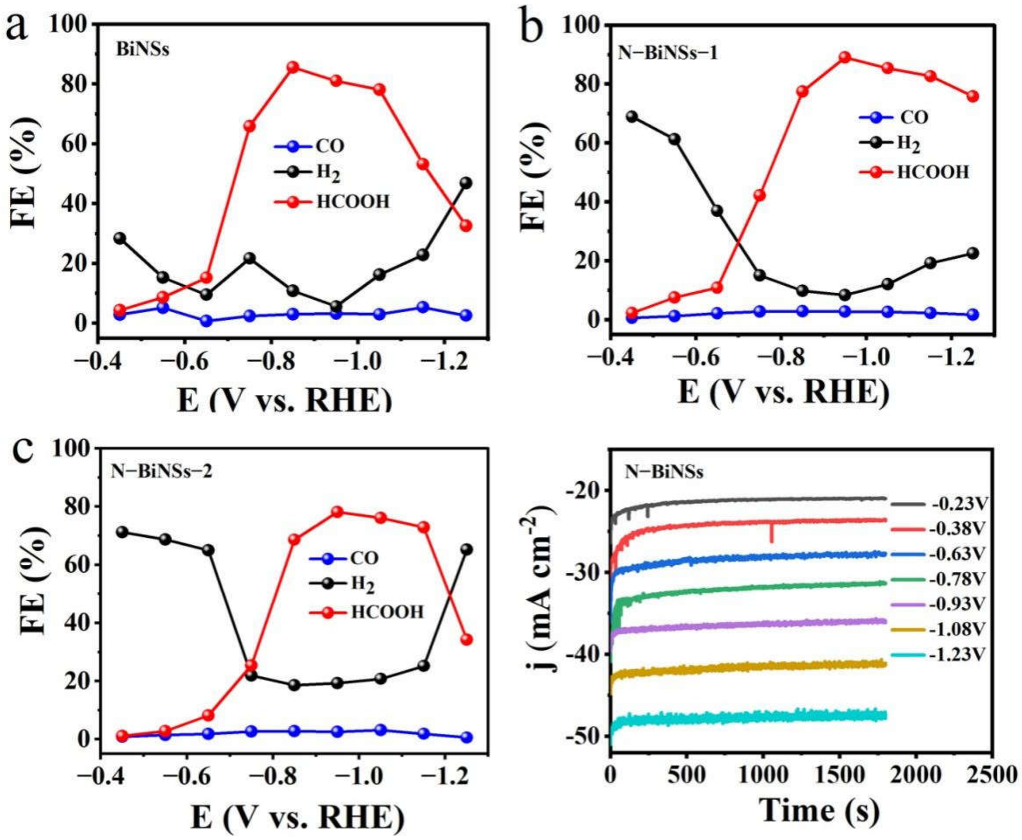

Figure S4. Total FE at different potentials in an H-type electrolytic cell (0.5 M $KHCO_3$) of (a) BiNSs, (b) N−BiNSs−1 (c) N−BiNSs−2 ; (d) Current response of N−BiNSs in 0.5 M $KHCO_3$ saturated with CO_2.

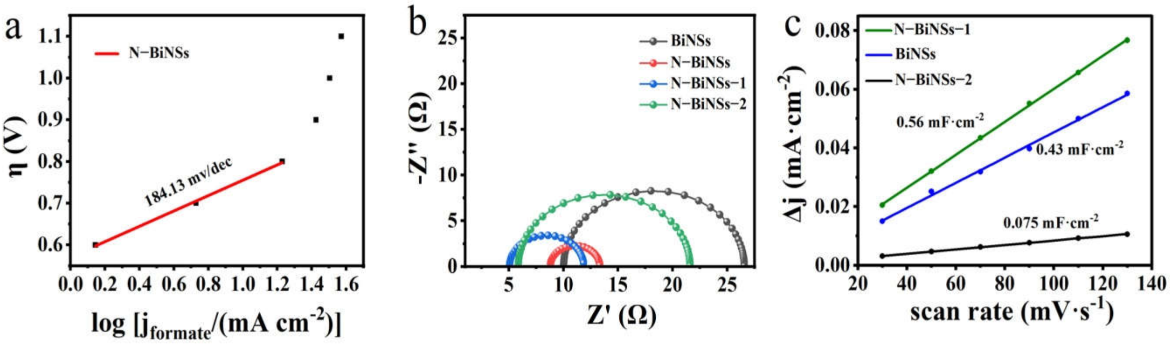

Figure S5. (a) Tafel slope test curve of N-BiNSs; (b) The impedance spectra of the catalyst at CP electrode were obtained under CO_2RR (-0.95 V vs. RHE) condition (frequency range: 10 kHz-1.0 Hz); (c) The BiNSs, N−BiNSs−1 and N−BiNSs−2 the relationship between charge current density difference (△J) and scanning rate.

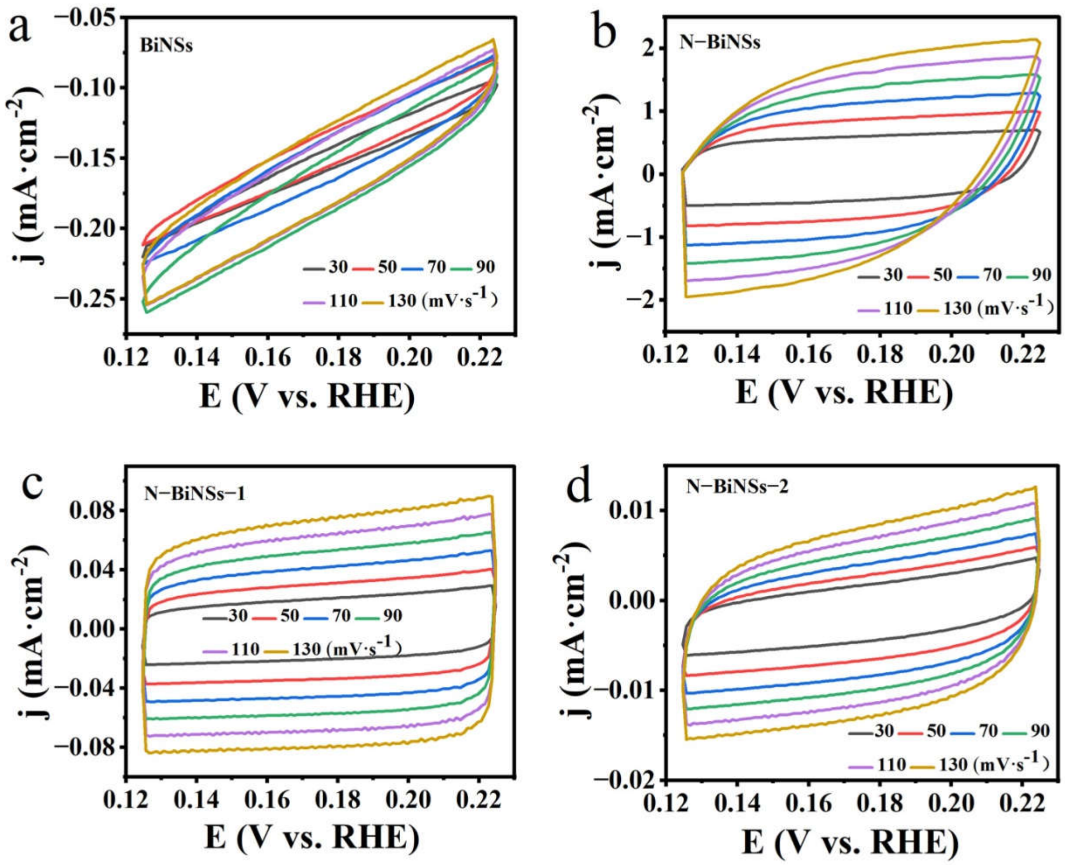

Figure S6. The catalyst (a) BiNS, (b) N−BiNSs, (c) N−BiNSs−1, and (d) N−BiNSs−2 cycle volt-ampere curve.

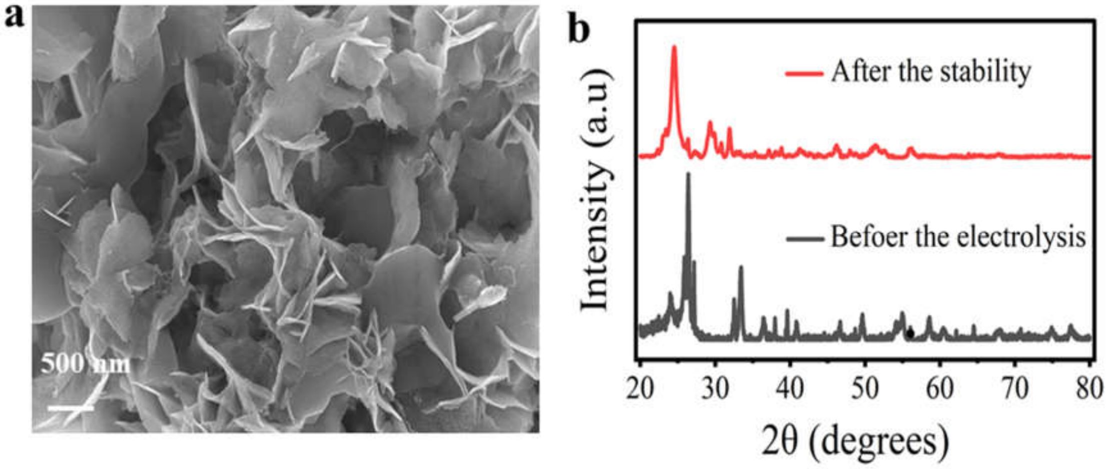

Figure S7. Total FE_{toal} different potentials in an H-type electrolytic cell (0.5 M $KHCO_3$) of (a) N−BiNSs, (a)SEM, (b) XRD.

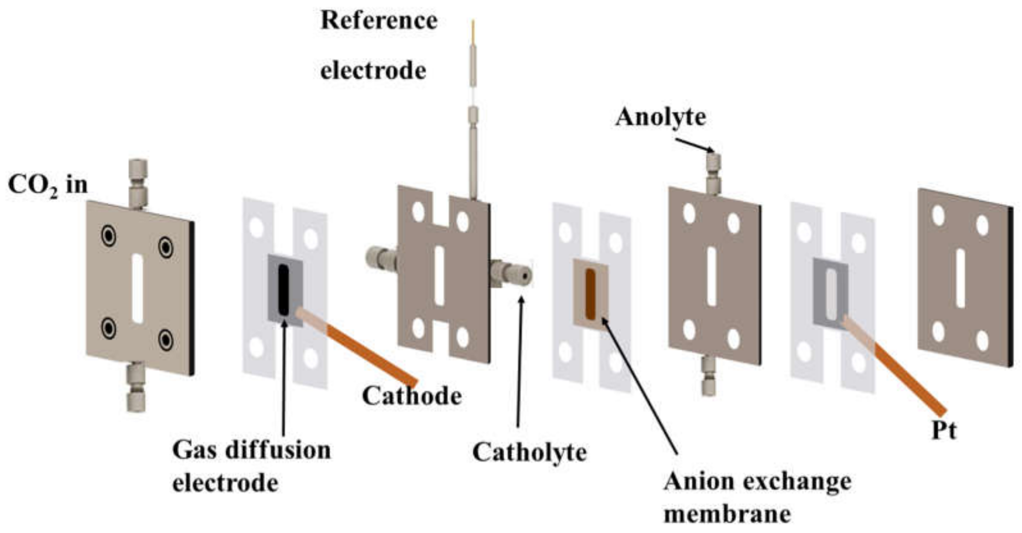

Figure S8. Schematic diagram of flow cell device used in electrocatalytic test.

References

1. Kresse, G.; Furthmuller, J., Efficient iterative schemes for ab initio total-energy calculations using a plane-wave basis set. *Phys Rev B Condens Matter.* 1996, 54, (16), 11169-11186.
2. Kresse, G.; Hafner, J., Ab initio molecular-dynamics simulation of the liquid-metal-amorphous-semiconductor transition in germanium. *Phys Rev B Condens Matter.* 1994, 49, (20), 14251-14269.
3. Blochl, P. E., Projector augmented-wave method. *Phys Rev B Condens Matter.* 1994, 50, (24), 17953-17979.
4. Perdew, J. P.; Burke, K.; Ernzerhof, M., Generalized Gradient Approximation Made Simple. *Phys Rev Lett*. 1996, 77, (18), 3865-3868.
5. Zhang, Y.; Yang, W., Comment on "Generalized Gradient Approximation Made Simple". *Physical Review Letters* 1998, 80, (4), 890.
6. Hammer, B.; Hansen, L. B.; Nørskov, J. K., Improved adsorption energetics within density-functional theory using revised Perdew-Burke-Ernzerh of functionals. *Physical Review B* 1999, 59, (11), 7413-7421.
7. Monkhorst, H. J.; Pack, J. D., Special points for Brillouin-zone integrations. *Physical Review B* 1976, 13, (12), 5188-5192.
8. Nørskov, J. K.; Rossmeisl, J.; Logadottir, A.; Lindqvist, L.; Kitchin, J. R.; Bligaard, T.; Jónsson, H., Origin of the Overpotential for Oxygen Reduction at a Fuel-Cell Cathode. *The Journal of Physical Chemistry B* 2004, 108, (46), 17886-17892.
9. Zhang, W.; Hu, Y.; Ma, L.; Zhu, G.; Zhao, P.; Xue, X.; Chen, R.; Yang, S.; Ma, J.; Liu, J.; Jin, Z., Liquid-phase exfoliated ultrathin Bi nanosheets: Uncovering the origins of enhanced electrocatalytic CO_2 reduction on two-dimensional metal nanostructure. *Nano Energy* 2018, 53, 808-816.
10. Xing, Y.; Kong, X.; Guo, X.; Liu, Y.; Li, Q.; Zhang, Y.; Sheng, Y.; Yang, X.; Geng, Z.; Zeng, J., Bi@Sn Core-Shell Structure with Compressive Strain Boosts the Electroreduction of CO_2 into Formic Acid. *Adv Sci.* 2020, 7, (22), 1902989.
11. Kwon, I. S.; Debela, T. T.; Kwak, I. H.; Seo, H. W.; Park, K.; Kim, D.; Yoo, S. J.; Kim, J.-G.; Park, J.; Kang, H. S., Selective electrochemical reduction of carbon dioxide to formic acid using indium–zinc bimetallic nanocrystals. *Journal of Materials Chemistry A* 2019, 7, (40), 22879-22883.
12. Cheng, Y.; Hou, J.; Kang, P., Integrated Capture and Electroreduction of Flue Gas CO_2 to Formate Using Amine Functionalized SnOx Nanoparticles. *ACS Energy Letters* 2021, 6, (9), 3352-3358.
13. Wu, Z.; Wu, H.; Cai, W.; Wen, Z.; Jia, B.; Wang, L.; Jin, W.; Ma, T., Engineering Bismuth-Tin Interface in Bimetallic Aerogel with a 3D Porous Structure for Highly Selective Electrocatalytic CO_2 Reduction to HCOOH. *Angew Chem Int Ed*. 2021, 60, (22), 12554-12559.
14. Li, F.; Xue, M.; Li, J.; Ma, X.; Chen, L.; Zhang, X.; MacFarlane, D. R.; Zhang, J. J. A. C., Unlocking the electrocatalytic activity of antimony for CO_2 reduction by two-dimensional engineering of the bulk material. *Angew Chem Int Ed*. 2017, 129, (46), 14910-14914.
15. Watanabe, M.; Shibata, M.; Kato, A.; Azuma, M.; Sakata, T. J. J. o. t. E. S., Design of alloy electrocatalysts for CO_2 reduction: III. The selective and reversible reduction of on Cu alloy electrodes. *Journal of The Electrochemical Society* 1991, 138, (11), 3382.
16. Sreekanth, N.; Nazrulla, M. A.; Vineesh, T. V.; Sailaja, K.; Phani, K. L., Metal-free boron-doped graphene for selective electroreduction of carbon dioxide to formic acid/formate. *Chem. Commun.* 2015, 51, (89), 16061-16064
17. Won, D. H.; Choi, C. H.; Chung, J.; Chung, M. W.; Kim, E. H.; Woo, S. I. J. C., Rational design of a hierarchical tin dendrite electrode for efficient

electrochemical reduction of CO_2. *ChemSusChem* 2015, 8, (18), 3092-3098.
18. Zheng, X.; De Luna, P.; García de Arquer, F. P.; Zhang, B.; Becknell, N.; Ross, M. B.; Li, Y.; Banis, M. N.; Li, Y.; Liu, M.; Voznyy, O.; Dinh, C. T.; Zhuang, T.; Stadler, P.; Cui, Y.; Du, X.; Yang, P.; Sargent, E. H., Sulfur-modulated tin sites enable highly selective electrochemical reduction of CO_2 to formate. *Joule* 2017, 1, (4), 794-805.
19. He, S.; Ni, F.; Ji, Y.; Wang, L.; Wen, Y.; Bai, H.; Liu, G.; Zhang, Y.; Li, Y.; Zhang, B.; Peng, H., The p-Orbital delocalization of main‐group metals to boost CO_2 electroreduction. *Angew Chem Int Ed*. 2018, 130, (49), 16346-16351.
20. Kumar, B.; Atla, V.; Brian, J. P.; Kumari, S.; Nguyen, T. Q.; Sunkara, M.; Spurgeon, J. M., Reduced SnO_2 porous nanowires with a high density of grain boundaries as catalysts for efficient electrochemical CO_2‐into‐HCOOH conversion. *Angew Chem Int Ed*. 2017, 56, (13), 3645-3649.
21. Liang, C.; Kim, B.; Yang, S.; Yang Liu, Y. L.; Francisco Woellner, C.; Li, Z.; Vajtai, R.; Yang, W.; Wu, J.; Kenis, P. J. A.; Ajayan, Pulickel M., High efficiency electrochemical reduction of CO_2 beyond the two-electron transfer pathway on grain boundary rich ultra-small SnO_2 nanoparticles. *Journal of Materials Chemistry A* 2018, 6, (22), 10313-10319.
22. Lee, C. W.; Hong, J. S.; Yang, K. D.; Jin, K.; Lee, J. H.; Ahn, H.-Y.; Seo, H.; Sung, N.-E.; Nam, K. T., Selective Electrochemical Production of Formate from Carbon Dioxide with Bismuth-Based Catalysts in an Aqueous Electrolyte. *ACS Catal*. 2018, 8, 931–937.
23. Zhang, Y.; Li, F.; Zhang, X.; Williams, T.; Easton, C. D.; Bond, A. M.; Zhang, J., Electrochemical reduction of CO_2 on defect-rich Bi derived from Bi_2S_3 with enhanced formate selectivity. *Journal of Materials Chemistry A* 2018, 6, (11), 4714-4720.
24. Zhao, Y.; Liang, J.; Wang, C.; Ma, J.; Wallace, G. G., Tunable and efficient tin modified nitrogen‐doped carbon nanofibers for electrochemical reduction of aqueous carbon dioxide. *Adv. Energy Mater*. 2018, 8, (10), 1702524.
